# Avalanche-size distribution of Cayley tree

**DOI:** 10.1038/s41598-023-38332-1

**Published:** 2023-07-13

**Authors:** Amikam Patron

**Affiliations:** Department of Mathematics, Jerusalem College of Technology, 91160 Jerusalem, Israel

**Keywords:** Complex networks, Phase transitions and critical phenomena

## Abstract

Attacks on networks is a very important issue in developing strategies of eradicating spreads of malicious phenomena in networks, such as epidemics and fake information. This field of research is referred to as *networks immunization*. The traditional approach to evaluating the effectiveness of attacks on networks focuses on measuring macro parameters related to the entire attack, such as the critical probability of a percolation occurrence in the network $p_c$ and the relative size of the largest component in the network, known as the *giant component*, but not considering the attack on a micro perspective, which is the analysis of node removals, during an attack, themselves, their characteristics and results. In this paper we present and apply the last method of focusing on the micro scale of an attack. Based on the theory of percolation in networks, we analyze the phenomenon of an *avalanche* which results due to a single node removal from a network. An avalanche is a state in which a removal of a single node from the giant component of a network leads to the disconnection of additional nodes. This process significantly contributes to the fragmentation (immunization) of the network, comparing to the impact of the initial node removal alone. Specifically, we focus on the *size* parameter of an avalanche, which is the number of nodes that are disconnected from the giant component due to a single node removal. Relating to a random attack on a network of the type of *Cayley tree*, we derive analytically the distribution of the sizes of avalanches that occur during the entire attack on it, until the network is dismantled (immunized) and the attack is terminated.

## Introduction

An attack on networks, which is a scenario where a combination of nodes (or links) of a network are removed and become non-functional causing the entire network to be dismantled and non-functional, is an issue that has been studied widely^1–23^. There are two types of attacks on networks, on them the research has been focused: *random attack* and *targeted attack*^1^. In a random attack, the attacker has no information about the network and its topology. Therefore, the choice of the nodes to be attacked is implemented randomly, with no priority of each node to be attacked before or after another node. In each attack stage, the attacker selects a node at random from the set of nodes that have not been attacked until that stage, and attacks it. On the other hand, in a targeted attack the attacker has some information about the network and its characteristics, and according to it there are some priorities of attacking some nodes before other nodes. Therefore, in each attack stage the attacker attacks the node with the highest priority among the set of nodes were not attacked until then. An example of a widely studied targeted attack is where the attacker has information about the degree of each node in the network, and at each stage of the attack the node with the highest degree among the nodes that have not been attacked yet, is selected to be attacked. Other traditional types of attacks on networks have also been studied, such as localized attack^2^ where the attack affects just a certain region of the network, removing nodes sequentially according to some node characteristics such as betweenness centrality^3^, a combination of the degree and betweenness centrality^4^, second neighbors degree^5^ and so on.

A very important issue related to attacks on networks, is the *robustness* of a network, which refers to its capability to remain functional during an attack. There have been many studies about the way of parameterizing the robustness of networks. Some approaches are based on comparisons of network’s parameters before and after the attack such as a change in the *network diameter*^6–8^, the relative size of the large component of the network^9^, change in the *betweeness centrality*^3^, *connectivity loss*^10^, and *f-robustness*^11^. Other methods are based on measuring the mean functionality level of a network during an entire attack such as the integral of the network’s largest component with respect to time during the attack^12–15^.

An important method of measuring the robustness of a network is based on *percolation theory*^24,25^. The basic description of percolation theory is related to lattices. It presents a state where the sites (or the bonds) of a lattice are occupied with probability *p*, and unoccupied with probability $1-p$. For each lattice structure (such as BCC, FCC, Honeycomb etc.) there is a critical probability denoted by $p_c$. If $p>p_c$, a cluster of occupied sites that spans the lattice from one side to the other, named *spanning cluster* or *infinite cluster*, exists in the lattice. Conversely, if $p<p_c$ there is no spanning cluster in the lattice^24^. When a spanning cluster exists in a lattice, we say that a *percolation* occurs in the lattice.

The same state could be presented in networks, where the nodes (or the edges) of a network are occupied with probability *p* and unoccupied with probability $1-p$. However, for defining a percolation in random networks, where the linking of the set of nodes by edges is performed randomly, we cannot apply the criterion of a spanning cluster as in lattices, since due to the network structure there is no meaning of spanning the network from one side to the other. Instead, another criterion has been stated for percolation in random networks, which is the existence of a large component of occupied nodes whose size scales with the network’s size, i.e. *O*(*N*), named *giant component*. That is, a typical property of a random network is a critical probability of node occupation $p_c$, such that if $p>p_c$, a giant component exists in the network, and if $p<p_c$, the network is fragmented into small components and a giant component does not exist. A smaller $p_c$ implies a more robust network, as it requires removing a large fraction of $1-p_c$ of the network’s nodes to fragment it.

In^16,17^ it was shown that the criterion for percolation in random network, i.e. the existence of a giant component in the network, was generated by the configuration model, is $\kappa =\frac{\langle k^2\rangle }{\langle k\rangle }>2$, where $\langle k\rangle$ is the expectation of the node degree and $\langle k^2\rangle$ is the expectation of the square of the node degree. According to this criterion, it was shown that for a random network under random attack

1 $$\begin{aligned} p_c=\frac{1}{\kappa _0-1}, \end{aligned}$$

where $\kappa _0$ is the value of $\kappa$ before the attack on the network begun^17^. From this, the value of $p_c$ for two types of random networks are studied widely, was calculated: (i) For Erdős–Rényi networks^18,19^, where the node’s degree *k* follows a Poisson distribution $P(k)=e^{-\lambda }\frac{\lambda ^k}{k!}$, it was shown that $p_c=\frac{1}{\lambda }$. (ii) For Scale-Free networks, a topology that was found in many real networks^20–23^, where the node’s degree *k* follows a power-law distribution $P(k)\sim k^{-\gamma }$, such that most of the nodes have a very small degree but there is also a small fraction of nodes with a very high degree named *hubs*, it was shown that for $\gamma >3$, $p_c$ has a finite nonzero value, but for $\gamma \le 3$, $p_c$ approaches 0 as *N* tends to infinity. That means that although almost all of the network’s nodes are removed, there still exists a giant component in the network and the network is considered functional.

Another type of network that has been studied widely including its robustness and aspects of percolation, is the Bethe-Lattice (BL). The BL is a network of the type of an infinite tree (no cycles) where all its nodes have the same degree *Z*. Using Eq. (1), it was shown^1^ that under random attack, the critical percolation threshold $p_c$ of the BL with *Z* neighbors per node is

2 $$\begin{aligned} p_c=\frac{1}{Z-1}. \end{aligned}$$

This expression was also derived in^26^ and in^27^, applying other methods. Another type of network that is similar to the BL but not identical is the Cayley tree (CT), which is a BL but with a finite number of nodes arranged in a finite number of layers. That is, in a CT there is a unique node from it the tree begins. This node is linked to *Z* neighbors that form the first layer of the tree. Then each node of the first layer is linked to other $Z-1$ neighbors, all of which form the second layer of the tree. Each node of the second layer is linked to other $Z-1$ neighbors, all of which form the third layer of the tree, and so on. This process is terminated in the last layer, where each node of this layer is not branched again to some other $Z-1$ nodes of some next layer. The critical probability $p_c$ of the CT is not the same as of BL, as its *finite-size* needs to be considered^24^. Below in this paper, we present a method for calculating the critical probability $p_c$ of a CT.

In general, the robustness of a network is a critical characteristics for its survivability under malicious attacks. However, there are typical states where an attack on a network is desirable, such as when an extinction of negative phenomena spreading within the network is required, as in the case of an epidemic, fake information, or damage. There are indeed many studies that address the issue of eradicating negative phenomena in networks^28–31^, which is known as *network immunization*.

However, almost all the studies on network attacks from the perspective of percolation theory, focus on the network’s macro-level properties, such as the percolation threshold $p_c$ and the size of the giant component relative to the network’s size. They do not address the micro-scale aspects of the attack, such as the characteristics and outcomes of individual node removals.

In this paper, we present the approach of analyzing attacks on networks from a micro-scale perspective, as mentioned above. Our focus is on a phenomenon called an *avalanche*, which occurs when an attack on a node that is part of the giant component causes it to disconnect from the component. Consequently, this disconnection, causes other nodes that were not directly attacked but are dependent on the attacked node to be disconnected from the giant component as well. The total number of nodes that are disconnected from the giant component due to an avalanche, is called the *size* of the avalanche. Specifically, we focus on a network of the CT type and develop analytical expressions for the probability distribution of the various sizes of avalanches that occur during a random attack on this network.

We note that in various studies, certain phenomena have been modeled using a series of avalanches on BL and CT, such as avalanches of an Abelian sandpile^32^, avalanches in a depinning model that model the growth of rough interfaces^33^, avalanches of occupied sites modeled by queuing models^34^, and avalanches of a gas during its propagation in the opened alveolus of the lung^35,36^. However, the model and analysis of avalanches that occur during an attack on a CT has not been studied yet, which is also an innovative aspect of this work.

## The model

We define the CT structure as follows: The node from it the tree begins is referred to as the ‘root’. A ‘neighbor’ of a node is a node that is linked to it. Moving away from the root, going from a node to its neighbor is called ‘walking down the tree’, while moving towards the root is called ‘walking up the tree’. The ‘sons’ of a node are the node’s neighbors when walking down the tree. The ‘father’ of a node is the node’s neighbor when walking up the tree. The ‘descendants’ of a node are all the nodes that can be reached from it by walking down the tree (a node’s son is also its descendant). The ‘ancestors’ of a node are all the nodes that can be reached from it by walking up the tree (a node’s father is also its ancestor). The first layer of the tree is the set of all the sons of the root and is referred to as ‘generation 1’. The second layer of the tree is the set of all the sons of nodes in generation 1 and is called ‘generation 2’, and so on. The number of layers in the tree is represented by *L*. The nodes in the *L*’th layer of the tree are the terminal nodes and are called the ‘surface’ of the tree. The set of these nodes is referred to as ‘generation *L*’. The network is attacked in stages, where each stage involves selecting a random node in the network to be attacked. Once a node has been attacked, it cannot be selected again. The attack continues until a sufficient number of nodes have been attacked, to cause a phase transition where the network is dismantled and the giant component is fragmented.

During an attack on the network, a node can be in one of three modes: *white*, *black*, or *grey*. Initially, before the attack begins, all nodes are considered functional and are in the *white* mode. During the attack, if a white node is attacked, it becomes non-functional and is switched to the *black* mode. Additionally, all its descendants that are still functional and in the *white* mode are switched to the non-functional *grey* mode, as the attacked node cuts off their path up the tree to the root. It is important to note that grey nodes, although non-functional, have not been attacked and can still be chosen to be attacked in subsequent stages. If a grey node is attacked, it is switched to the *black* mode. According to these definitions of the three modes, it is guaranteed that the ancestors of a white node can be only white, and the descendants of a white node can be white or black. The ancestors of a grey node can be grey or black, and the descendants of a grey node can be grey or black. The ancestors of a black node can be white, grey, or black, and the descendants of a black node can be grey or black. Since the root of the tree has no father, it can be in white mode or black mode only.

‘Avalanche’ as defined above is a combination of nodes that become disconnected from the giant component due to the removal of a single node. In our model, an avalanche is a change in the mode of a set of white nodes resulting from an attack on a white node. The attacked node switches to black mode, and its white descendants switch to grey mode. The ‘avalanche size’ is the number of white nodes that change their mode during an avalanche. A ‘complete avalanche’ is an avalanche where all the descendants of the white attacked node are also white. This causes the attacked white node to switch to a black mode, and all its descendants down the tree until the surface of the tree switched to a grey mode. For example, in a CT with *Z* neighbors, attacking a white node in the *L*th layer causes a complete avalanche of size 1. Attacking a white node in the $L-1$ layer, where its $Z-1$ sons of the *L*th layer are in white mode, causes a complete avalanche of size $1+(Z-1)=\frac{(Z-1)^2-1}{Z-2}=Z$. Attacking a white node in the $L-2$ layer, where its $Z-1+\left( Z-1\right) ^2$ descendants ($Z-1$ of the $L-1$ layer and $\left( Z-1\right) ^2$ of the *L*th layer) are in white mode, causes a complete avalanche of size $1+(Z-1)+(Z-1)^2=\frac{(Z-1)^3-1}{Z-2}$, and so on. In general, attacking a white node in the $L-m+1$ layer ($m=1,2,3,\ldots ,L$), where all its descendants are in white mode, causes a complete avalanche of size $\frac{(Z-1)^m-1}{Z-2}$. An ‘incomplete avalanche’ is an avalanche that is not of the complete avalanche type. It occurs when the descendants of a white attacked node are not all in white mode (some of them were switched to a black or a grey mode before the current attack on their ancestor). According to the formal definition of an avalanche, attacking a grey node does not cause an avalanche (since it was already disconnected from the giant component before it was attacked). However, we define this state as a ‘null avalanche,’ meaning that a node was attacked but it does not contribute to the reduction of the giant component size. The null avalanche size is 0.

**Figure 1:**
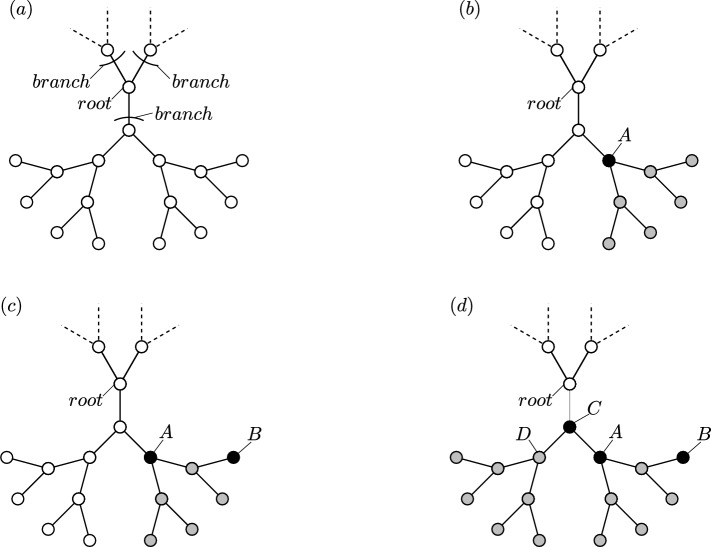
Illustration of the model on a Cayley tree with $Z=3$ neighbors and $L=3$ layers: (**a**) CT before an attack begins. All the nodes are in white mode. The *root* of the tree and the three *branches* emanating from the root are marked in the sketch. For clarity, only the nodes belonging to the bottom branch are depicted. (**b**) Stage one of an attack—Node *A* is attacked. As a result, node *A* switches to the black mode, and its six descendants switch to the grey mode. Prior to this stage, all descendants of node *A* were in the white mode, so the attack on node *A* causes a *complete avalanche* of size 7 (node *A* and its six descendants). (**c**) Stage two of an attack—Node *B* is attacked. As a result, it is switches to the black mode. Since node *B* was in the grey mode, indicating disconnection from the root before this stage, the attack on node *B* leads to a *null avalanche*. (**d**) Stage three of an attack—Node *C* is attacked. Consequently, it is switches to the black mode. Since node *A* which is a *son* of node *C* was already attacked prior to this stage, the current stage has no impact on that sub-branch containing node *A* and its descendants. On the other hand, the nodes in the other sub-branch of node *C*, including node *D* and its descendants, which were in the white mode before this stage, all switch to the grey mode. As not all descendants of node *C* were in the white mode before this stage, the current stage results in an *incomplete avalanche* of size 8 (node *C*, node *D* and node *D*’s six descendants).

Figure 1 is an illustration of the model. It presents three stages of an attack on a CT with $Z=3$ neighbors and $L=3$ layers, including presentations of a complete avalanche, an incomplete avalanche and a null avalanche that occur during the attack.

## Critical probability $p_c$ of a Cayley tree

I. Definitions.

We define the following regarding random choices of nodes in the tree: the probability of randomly selecting a black node is denoted by $p_b$. Since only one node is attacked and switched to a black mode at each attack stage, then between stages *k* and $k+1$ of an attack, $p_b=\frac{k}{N}$, where *N* is the size of the tree.

We denote the events of randomly selecting a node of white, grey or black mode as *W*, *G* and *B*, respectively. The events of selecting a node that is not of white, not of grey, or not of black mode are denoted as $\overline{W}$, $\overline{G}$ and $\overline{B}$, respectively. The events of randomly selecting a node of the first generation, second generation, and so on until the *L*’th generation are denoted as $g_1$, $g_2$, and so on until $g_L$, respectively.

II. Critical probability $p_c$.

A defining property of a CT, resulting from its structure as a tree with no cycles, is that there is only one path from any node to the root. Therefore, percolation in a CT is defined as a state in which there is at least one path from a node on the surface of the tree to the root consisting of functional nodes, i.e., nodes of white mode only. As mentioned earlier, according to our model, descendants of nodes in grey or black mode could be of grey or black mode only, but not of white mode. This guarantees that for each node in white mode, all of its ancestors until the root are also in white mode. Specifically, the presence of a white node on the surface of the tree guarantees a path from it to the root composed of white nodes only, fulfilling the percolation condition. Therefore, to calculate $p_c$ of a CT, we first calculate the probability of randomly selecting a white node, given that it belongs to the surface of the tree. We perform the calculation in several stages, as follows: For the first generation—the event of selecting a node that is not in a white mode, given that this node is of the first generation, occurs when the father of this node (i.e., the root) is in a black mode, with probability $p_b$, or when the chosen node itself is in a black mode and its father (i.e., the root) is in a white mode, with probability $p_b\left( 1-p_b\right)$. Thus, the probability of the complementary event of choosing a white node, given that this node is of the first generation, is

3 $$\begin{aligned} P\left( W|g_1\right) =1-\left[ p_b+p_b\left( 1-p_b\right) \right] =\left( 1-p_b\right) ^2. \end{aligned}$$

Similarly for the second generation—the event of selecting a node that is in a non-white node given that it is of the second generation occurs when the ancestor of distance 2 of that node, i.e. the root, is black with probability $p_b$, or when the father of that node is black and its ancestor of distance 2 is white with probability $p_b\left( 1-p_b\right)$, or when the chosen node itself is black and both its father and its ancestor of distance 2 are white with probability $p_b\left( 1-p_b\right) ^2$. Thus, the probability of the complement event of choosing a white node given that it is of the second generation is

4 $$\begin{aligned} P\left( W|g_2\right) =1-\left[ p_b+p_b\left( 1-p_b\right) +p_b\left( 1-p_b\right) ^2\right] =\left( 1-p_b\right) ^3. \end{aligned}$$

The above results can be generalized to any generation denoted by *l*, such that the probability of selecting a node that is white given that this node is of the *l*-th generation is

5 $$\begin{aligned} P\left( W|g_l\right) =\left( 1-p_b\right) ^{l+1}, \end{aligned}$$

and in particular, the probability of selecting a white node given that it belongs to the tree surface, i.e., it belongs to the *L*-th generation, is $\left( 1-p_b\right) ^{L+1}$.

In a CT with *Z* neighbors, the number of nodes of the surface is $Z\left( Z-1\right) ^{L-1}$. We number the nodes of the surface with the numbers $1, 2, 3, \ldots , Z\left( Z-1\right) ^{L-1}$. Let us define an indicator random variable $X_j$ for the event of the existence of a path from the node of the surface numbered *j* to the root, consisting only of white nodes. That is, $X_j$ represents the event of percolation due to the *j*-th node on the surface. We also define another random variable *X* that represents the number of paths from the surface to the root consisting only of white nodes. Therefore, we have $X = \sum _{j=1}^{Z\left( Z-1\right) ^{L-1}}X_j$. The expected number of such paths from the surface to the root can be calculated as

6 $$\begin{aligned} E\left[ X\right]&=\sum _{j=0}^{Z\left( Z-1\right) ^{L-1}}E\left[ X_j\right] =\sum _{j=0}^{Z\left( Z-1\right) ^{L-1}}\left( 1-p_b\right) ^{L+1}\nonumber \\&=Z\left( Z-1\right) ^{L-1}\left( 1-p_b\right) ^{L+1}. \end{aligned}$$

The condition for percolation is that the expected number of such paths from the surface to the root is at least 1, i.e., $E\left[ X\right] \ge 1$. The transition occurs when $E\left[ X\right] =1$. Therefore, we define $p_b^*$ as the critical value of $p_b$ at the transition, and obtain

7 $$\begin{aligned} Z\left( Z-1\right) ^{L-1}\left( 1-p_b^*\right) ^{L+1}=1. \end{aligned}$$

Thus, the critical probability $p_c$ which is the fraction of nodes that were not attacked at the critical point of percolation can be expressed as

8 $$\begin{aligned} p_c=1-p_b^*=\frac{\left( Z-1\right) ^\frac{1-L}{1+L}}{Z^\frac{1}{1+L}}. \end{aligned}$$

Note that when $L>>1$, $p_c$ in Eq. (8) tends to be approximated by

9 $$\begin{aligned} p_c=\frac{1}{Z-1}, \end{aligned}$$

which is the known percolation condition of BL. Therefore, we conclude that when $L>>1$, the critical probability $p_c$ of a CT tends to be the same as that of the BL.

In this paper we analyze CTs with a very large *L*, so we consider Eq. (9) as the critical probability for percolation in CTs.

## Avalanche-size distribution of a Cayley tree with *Z* neighbors per node

### Theory

#### Probability of a null avalanche

I. Probability of choosing a grey node.

Recall that a null avalanche occurs when a grey node is attacked, and that black nodes that have already been attacked are not chosen to be attacked again. Therefore, we first calculate the probability of randomly selecting a grey node given that the chosen node is not black. To calculate this probability, we first perform a simpler calculation of the probability of selecting a white node at random given that the chosen node is not black. We do this in stages as follows: for the first generation—the event of selecting a node that is not white given that this node is of the first generation and is not black occurs when the father of this node (the root) is black, and its probability is $p_b$. Thus, the probability of the complement event of selecting a white node given that this node is of the first generation and is not black is

10 $$\begin{aligned} P\left( W|g_1\cap \overline{B}\right) =1-p_b. \end{aligned}$$

Similarly, for the second generation, the event of selecting a node that is not white given it is of the second generation and is not black occurs when the ancestor of distance 2 of this node (the root) is black, with probability $p_b$, or when the father of this node is black and its ancestor of distance 2 is white, with probability $p_b\left( 1-p_b\right)$. Thus, the probability of the complement event of selecting a node that is white given that this node is of the second generation and is not black is

11 $$\begin{aligned} P\left( W|g_2\cap \overline{B}\right) =1-\left[ p_b+p_b\left( 1-p_b\right) \right] =\left( 1-p_b\right) ^2. \end{aligned}$$

Similarly, the previous results can be generalized for any generation *l*, such that the probability of selecting a node that is white given that this node is of the *l*-th generation and is not black is

12 $$\begin{aligned} P\left( W|g_l\cap \overline{B}\right) =\left( 1-p_b\right) ^l. \end{aligned}$$

As a chosen node can only be white or grey, the probability of selecting a grey node that is in the *l*’th generation and is not black is the complement of the probability of selecting a white node with those same characteristics. Therefore, we have

13 $$\begin{aligned} P\left( G|g_l\cap \overline{B}\right) =1-\left( 1-p_b\right) ^l. \end{aligned}$$

Using the total probability formula, we can calculate the probability of selecting a grey node in the *l*’th generation that is not black as follows:

14 $$\begin{aligned} P\left( G\cap g_l|\overline{B}\right) =P\left( G|g_l\cap \overline{B}\right) \cdot P\left( g_l|\overline{B}\right) . \end{aligned}$$

Since the randomness of selecting the nodes to be attacked causes independence of the events of choosing a node of some generation and of choosing a node that is not black, we have $P(g_l|\overline{B})=P(g_l)$, which is the probability of choosing a node of the *l*’th generation. This probability is the ratio between the number of nodes in the *l*’th generation, which for a CT with *Z* neighbors is $Z\cdot (Z-1)^{l-1}$, and the total number of nodes in the tree, which for a CT with *Z* neighbors is $\frac{1}{Z-2}[Z(Z-1)^L-2]$. Assuming a very large *L*, this ratio is approximately $\left( Z-2\right) \left( Z-1\right) ^{-\left( L-l+1\right) }$. Thus, we get

15 $$\begin{aligned} P\left( G\cap g_l|\overline{B}\right) =\left[ 1-\left( 1-p_b\right) ^l\right] \cdot \left( Z-2\right) \left( Z-1\right) ^{-\left( L-l+1\right) }. \end{aligned}$$

Following this result, we get that the probability of selecting a grey node given that it is not black, without considering the generation to which the node belongs, is given by:

16 $$\begin{aligned} P\left( G|\overline{B}\right)&=\sum _{l=1}^{L}\left[ 1-\left( 1-p_b\right) ^l\right] \cdot \left( Z-2\right) \left( Z-1\right) ^{-\left( L-l+1\right) }\nonumber \\&=\left( Z-2\right) \left( Z-1\right) ^{-\left( L+1\right) }\nonumber \\&\quad \cdot \left[ \sum _{l=1}^{L}\left( Z-1\right) ^l-\sum _{l=1}^{L}\left[ \left( Z-1\right) \left( 1-p_b\right) ^l\right] \right] . \end{aligned}$$

We calculate the series and neglect some terms due to the assumption of a very large *L*, and obtain:

17 $$\begin{aligned} P\left( G|\overline{B}\right) =1-\frac{\left( Z-2\right) \left( 1-p_b\right) ^{L+1}}{\left( Z-2\right) -\left( Z-1\right) p_b}. \end{aligned}$$

Since between the stages *k* and $k+1$ of an attack, $p_b=\frac{k}{N}$, we get for the probability of selecting grey node between these stages:

18 $$\begin{aligned} P\left( G|\overline{B}\right) =1-\frac{\left( Z-2\right) \left( 1-\frac{k}{N}\right) ^{L+1}}{\left( Z-2\right) -\left( Z-1\right) \frac{k}{N}}. \end{aligned}$$

II. Probability of a null avalanche during an attack.

Equation (18) can be interpreted as the probability that on stage $k+1$ of a random attack on the tree, a grey node is chosen to be attacked, i.e. the probability of the event of a null avalanche on stage $k+1$. We define an indicator random variable $X_k$ for the event of a null avalanche in stage $k+1$, and a random variable *X* that is the number of null avalanches during an entire attack until the network collapses. Recall that for a CT with a very large *L* and *Z* neighbors, $p_c=\frac{1}{Z-1}$ (Eq. (9) above), i.e. an attack on a fraction of $\frac{Z-2}{Z-1}$ of the network’s nodes is required in order to dismantle the network, therefore, $X=\sum _{k=0}^{\frac{Z-2}{Z-1}N-1}X_k$. Accordingly, the expected number of null avalanches during an attack until the network collapses is

19 $$\begin{aligned} E\left[ X\right]&=\sum _{k=0}^{\frac{Z-2}{Z-1}N-1}E\left[ X_k\right] \nonumber \\&=\sum _{k=0}^{\frac{Z-2}{Z-1}N-1}\left( 1-\frac{\left( Z-2\right) \left( 1-\frac{k}{N}\right) ^{L+1}}{\left( Z-2\right) -\left( Z-1\right) \frac{k}{N}}\right) \nonumber \\&=\frac{Z-2}{Z-1}N-\sum _{k=0}^{\frac{Z-2}{Z-1}N-1}\frac{\left( Z-2\right) \left( 1-\frac{k}{N}\right) ^{L+1}}{\left( Z-2\right) -\left( Z-1\right) \frac{k}{N}}. \end{aligned}$$

We approximate the last expression in the right-hand-side of Eq. (19), by the following integral

20 $$\begin{aligned} \int _{0}^{\frac{Z-2}{Z-1}N-1}\frac{\left( Z-2\right) \left( 1-\frac{k}{N}\right) ^{L+1}}{\left( Z-2\right) -\left( Z-1\right) \frac{k}{N}}dk. \end{aligned}$$

We will now calculate the integral in Eq. (20) using tools from combinatorics and probability theory. This will lead us to the final expression presented below in Eq. (30). The detailed calculations and explanations can be found in the Supplementary Information, but we present here only the general outlines.

By substituting $u=\left( Z-2\right) -\left( Z-1\right) \frac{k}{N}$ and performing some algebraic operations, the integral in Eq. (20) is transformed to the following integral

21 $$\begin{aligned} -\frac{\left( Z-2\right) N}{\left( Z-1\right) ^{L+2}}\int _{Z-2}^{\frac{Z-1}{N}}\frac{\left( 1+u\right) ^{L+1}}{u}du. \end{aligned}$$

Performing the binomial formula of $(1+u)^{L+1}=\sum _{m=0}^{L+1}{L+1 \atopwithdelims ()m}u^m$ yields

22 $$\begin{aligned} -\frac{\left( Z-2\right) N}{\left( Z-1\right) ^{L+2}}\sum _{m=0}^{L+1}{L+1 \atopwithdelims ()m}\int _{Z-2}^{\frac{Z-1}{N}}u^{m-1}du. \end{aligned}$$

Each value of *m* in the summation in Eq. (22) yields a definite integral in which the antiderivative of the integrand is a polynomial in *u*, except for $m=0$, which yields a definite integral of the integrand $\frac{1}{u}$ whose antiderivative is the natural logarithm of *u*. Therefore, the term for $m=0$ is negligible relative to the terms for $m\ne 0$, so we neglect it and calculate the summation for $m=1$ to $m=L+1$ only. Accordingly, by solving the integral in Eq. (22), we get

$$\begin{aligned} -\frac{\left( Z-2\right) N}{\left( Z-1\right) ^{L+2}}\sum _{m=1}^{L+1}{L+1 \atopwithdelims ()m}\frac{1}{m}\left[ \left( \frac{Z-1}{N}\right) ^m-\left( Z-2\right) ^m\right] , \end{aligned}$$

Since we assume a very large value for *N*, we can neglect the term $\left( \frac{Z-1}{N}\right) ^m$, which is much smaller than $\left( Z-2\right) ^m$. This leads to the following equation:

23 $$\begin{aligned} \frac{\left( Z-2\right) N}{\left( Z-1\right) ^{L+2}}\sum _{m=1}^{L+1}\left( Z-2\right) ^m{L+1 \atopwithdelims ()m}\frac{1}{m}. \end{aligned}$$

By substituting the approximated value of *N* for a CT with a very large number of layers *L*, which is $\frac{Z}{Z-2}\left( Z-1\right) ^L$, into Eq. (23), we get:

24 $$\begin{aligned} \frac{Z}{\left( Z-1\right) ^2}\sum _{m=1}^{L+1}\left( Z-2\right) ^m{L+1 \atopwithdelims ()m}\frac{1}{m}. \end{aligned}$$

Applying the binomial coefficient identity $\sum _{j=1}^{L+1}\frac{1}{j}{j \atopwithdelims ()m}={L+1 \atopwithdelims ()m}\frac{1}{m}$ to Eq. (24) gives:

25 $$\begin{aligned} \frac{Z}{\left( Z-1\right) ^2}\sum _{j=1}^{L+1}\frac{1}{j}\sum _{m=1}^{L+1}{j \atopwithdelims ()m}\left( Z-2\right) ^m. \end{aligned}$$

Since ${j \atopwithdelims ()m}=0$ when $m>j$, we determine the upper limit of the second summation in Eq. (25) to be *j* only. Also, $\left( Z-1\right) ^j=\sum _{m=0}^{j}{j \atopwithdelims ()m}\left( Z-2\right) ^m=1+\sum _{m=1}^{j}{j \atopwithdelims ()m}\left( Z-2\right) ^m$. Substituting it into Eq. (25) gives the following formula:

26 $$\begin{aligned} \frac{Z}{\left( Z-1\right) ^2}\sum _{j=1}^{L+1}\frac{\left( Z-1\right) ^j-1}{j}. \end{aligned}$$

Changing the variable *j* to $L-j+1$, applying the identity $\frac{1}{L-j+1}= \frac{1}{L+1}\sum _{m=0}^{\infty }\frac{j^m}{\left( L+1\right) ^m}$, and ignoring relatively small terms, approximates Eq. (26) to:

27 $$\begin{aligned} Z\left( Z-1\right) ^{L-1}\sum _{m=0}^{\infty }\frac{1}{\left( L+1\right) ^{m+1}}\sum _{j=0}^{\infty }\frac{j^m}{\left( Z-1\right) ^j}. \end{aligned}$$

Considering the size *N* of a CT with *Z* neighbors and a very large *L* that is $\frac{Z}{Z-2}\left( Z-1\right) ^L$, we have $Z\left( Z-1\right) ^{L-1}=\frac{Z-2}{Z-1}N$. Substituting it into Eq. (27) gives:

28 $$\begin{aligned} \frac{Z-2}{Z-1}N\sum _{m=0}^{\infty }\frac{1}{\left( L+1\right) ^{m+1}}\sum _{j=0}^{\infty }\frac{j^m}{\left( Z-1\right) ^j}. \end{aligned}$$

By applying the series $\sum _{j=0}^{\infty }\frac{j^0}{\left( Z-1\right) ^j}=\frac{Z-1}{Z-2}$, $\sum _{j=0}^{\infty }\frac{j^1}{\left( Z-1\right) ^j}=\frac{Z-1}{\left( Z-2\right) ^2}$, and $\sum _{j=0}^{\infty }\frac{j^2}{\left( Z-1\right) ^j}=\frac{Z\left( Z-1\right) }{\left( Z-2\right) ^3}$, we can obtain the first few terms of the asymptotic expansion of Eq. (28) as:

$$\begin{aligned}&\frac{Z-2}{Z-1}N\\& \quad \cdot \left( \frac{\frac{Z-1}{Z-2}}{L+1}+\frac{\frac{Z-1}{\left( Z-2\right) ^2}}{\left( L+1\right) ^2}+\frac{\frac{Z\left( Z-1\right) }{\left( Z-2\right) ^3}}{\left( L+1\right) ^3}+\mathcal {O}\left( \frac{1}{\left( L+1\right) ^4}\right) \right) , \end{aligned}$$

Simplifying the above expression by changing the base of the expansion to *L*, we get:

29 $$\begin{aligned} N\left( \frac{1}{L}+\frac{\frac{3-Z}{Z-2}}{L^2}+\frac{\frac{Z^2-5Z+8}{2\left( Z-2\right) ^2}}{L^3}+\mathcal {O}\left( \frac{1}{L^4}\right) \right) . \end{aligned}$$

Since we assume a very large *L*, we neglect the terms of $\mathcal {O}\left( \frac{1}{L^2}\right)$ in Eq. (29) and consider only the first term of $\frac{N}{L}$. Therefore, we obtain the following final approximate expression for the integral in Eq. (20):

30 $$\begin{aligned} \int _{0}^{\frac{Z-2}{Z-1}N-1}\frac{\left( Z-2\right) \left( 1-\frac{k}{N}\right) ^{L+1}}{\left( Z-2\right) -\left( Z-1\right) \frac{k}{N}}dk \approx \frac{N}{L}. \end{aligned}$$

Substituting Eq. (30) into Eq. (19) gives the expected number of null avalanches during an entire attack:

31 $$\begin{aligned} E\left[ X\right] =\frac{Z-2}{Z-1}N-\frac{N}{L}. \end{aligned}$$

Assume a random choice of one attack stage from the set of $\frac{Z-2}{Z-1}N$ attack stages. Let $S_0$ be the event of a null avalanche that occurs due to the node removal in the chosen stage. The probability of $S_0$ is the ratio of the expectation of the number of null avalanches during an entire attack *E*[*X*] (calculated above in Eq. (31)), and the number of attack stages $\frac{Z-2}{Z-1}N$. Therefore, we get

32 $$\begin{aligned} P\left( S_0\right) =1-\frac{\frac{Z-1}{Z-2}}{L}. \end{aligned}$$

#### Probability of a complete avalanche

I. Definitions.

We state the following definitions: for this section, the event of randomly selecting a node of white mode from the *l*-th generation is denoted as $W_l$. $W^1_l$ denotes the same event as $W_l$, with the added condition that the selected node is son no. 1 of its father. Similarly, $W^2_l$ denotes the event of selecting a node that is son no. 2 of its father, $W^3_l$ denotes the event of selecting a node that is son no. 3 of its father, and so on, until $W^{Z-1}_l$ denotes the event of selecting a node that is son no. $Z-1$ of its father.

A ‘complete cluster’ is a set of nodes consisting of a father and all of its descendants, where all of them are in white mode. The father is referred to as the ‘head’ of the complete cluster. If a father of a complete cluster is attacked, than a complete avalanche occurs. The event of randomly selecting a node that is a head of a complete cluster is denoted as *C*. $C^1$ denotes the same event as *C*, with the added condition that the selected node is son no. 1 of its father. Similarly, $C^2$ denotes the same event as *C* with the added condition that the selected node is son no. 2 of its father, $C^3$ denotes the event of selecting a node that is son no. 3 of its father, and so on, until $C^{Z-1}$ denotes the event of selecting a node that is son no. $Z-1$ of its father.

II. Conditional probability of a complete avalanche of size $\frac{\left( Z-1\right) ^m-1}{Z-2} \ (m=1,2,3,\ldots ,L)$ in the $k+1$ stage of an attack.

We define $q_l$ as the probability of randomly selecting a node that is a head of a complete cluster, given that this node is in white mode and is in the *l*th generation, i.e. $q_l=P(C|W_l)$.

For simplicity, we will develop the following principles for a CT with $Z=3$ neighbors, and later generalize the results to a CT with any number of neighbors *Z*. For a CT with $Z=3$, the probability $q_l$ can also be related to the events related to the two sons of the chosen node, as follows

33 $$\begin{aligned} q_l=P\left[ \left( C^1\cap W^1_{l+1}\right) \cap \left( C^2\cap W^2_{l+1}\right) |W_l\right] . \end{aligned}$$

Due to the independence of the sons, the previous expression can be written as follows:

34 $$\begin{aligned} q_l=P\left[ \left( C^1\cap W^1_{l+1}\right) |W_l\right] \cdot P\left[ \left( C^2\cap W^2_{l+1}\right) |W_l\right] . \end{aligned}$$

We will now develop the term $P\left[ \left( C^1\cap W^1_{l+1}\right) |W_l\right]$. According to the conditional probability formula $P\left( A\cap B|C\right) =P\left( A|B\cap C\right) \cdot P\left( B|C\right)$, we have:

35 $$\begin{aligned} P\left[ \left( C^1\cap W^1_{l+1}\right) |W_l\right] =P\left( C^1|W^1_{l+1}\cap W_l\right) \cdot P\left( W^1_{l+1}|W_l\right) . \end{aligned}$$

Since $W^1_{l+1}\subset W_l$, we can write this as follows:

36 $$\begin{aligned}&P\left( C^1|W^1_{l+1}\right) \cdot P\left( W^1_{l+1}|W_l\right) \nonumber \\&\qquad =q_{l+1}(1-p_b). \end{aligned}$$

Since $P\left[ \left( C^1\cap W^1_l\right) |W_l\right]$=$P\left[ \left( C^2\cap W^2_l\right) |W_l\right]$, we can write $q_l$ in Eq. (34) using the following recursion relation:

37 $$\begin{aligned} q_l=\left( 1-p_b\right) ^2 q_{l+1}^2, \end{aligned}$$

where $l=1,2,3,\ \ldots \,L-1,L$. When considering a complete cluster of size 1 that is a white node from the surface of the tree (i.e., the last tree layer *L*), the event of choosing a node that is a head of a complete cluster given that this node is of the *L*’th generation and of a white mode is a sure event with probability 1. Therefore, combining this with the recursion relation of Eq. (37), we also have the boundary condition $q_L=1$.

The recursion relation of Eq. (37) can be generalized to a CT with any number *Z* of neighbors by applying the same rationales as in Eqs. (33)–(36), which were presented for the case of $Z=3$. Thus, the recursion relation and boundary condition for the general case become

38 $$\begin{aligned}&q_l=\left( 1-p_b\right) ^{Z-1} q_{l+1}^{Z-1}.\nonumber \\&q_L=1. \end{aligned}$$

Using this relation, we can derive formulas for $q_{L-m+1}$ for any $m\ge 1$. For example,

$$\begin{aligned}&q_{L-1}=\left( 1-p_b\right) ^{Z-1}q_L^{Z-1}=\left( 1-p_b\right) ^{Z-1}\\&q_{L-2}=\left( 1-p_b\right) ^{Z-1}q_{L-1}^{Z-1}=\left( 1-p_b\right) ^{\left( Z-1\right) ^2+\left( Z-1\right) }, \end{aligned}$$

and in general

39 $$\begin{aligned} q_{L-m+1}&=\left( 1-p_b\right) ^{\sum _{j=1}^{m-1}\left( Z-1\right) ^j}\nonumber \\&=\left( 1-p_b\right) ^{\frac{1}{Z-2}\left( \left( Z-1\right) ^m-Z+1\right) }. \end{aligned}$$

Taking into consideration that between the stages *k* and $k+1$ of an attack $p_b=\frac{k}{N}$, we can derive a general formula for the probability of randomly attacking a node that is a head of a complete cluster at the $k+1$ stage of an attack, given that the node is of white mode and of the $L-m+1$ generation. This probability corresponds to the probability of attacking a node that causes a complete avalanche of size $\frac{\left( Z-1\right) ^m-1}{Z-2}$ at the $k+1$ stage of an attack, given that this node is of a white mode. Using the above expression, this probability can be expressed as

40 $$\begin{aligned} q_{L-m+1}=\left( 1-\frac{k}{N}\right) ^{\frac{1}{Z-2}\left( \left( Z-1\right) ^m-Z+1\right) }, \end{aligned}$$

where $$m=1,2,3,\ \ldots \,L$$.

III. Probability of a complete avalanche of size $\frac{\left( Z-1\right) ^m-1}{Z-2} \ (m=1,2,3,\ldots ,L)$ in the $k+1$ stage of an attack.

We define $Q_{L-m+1}$ as the probability of randomly selecting a node that is a head of complete cluster and is in a white mode and of generation $L-m+1$, given that the node is not black, i.e. $Q_{L-m+1}=P\left( C\cap W\cap g_{L-m+1}|\overline{B}\right)$. Applying the conditional probability formula, we get

41 $$\begin{aligned}&Q_{L-m+1}\nonumber \\&\quad =P\left( C|W\cap g_{L-m+1}\cap \overline{B}\right) \cdot P\left( W|g_{L-m+1}\cap \overline{B}\right) \cdot \nonumber \\&\qquad \cdot P\left( g_{L-m+1}|\overline{B}\right) . \end{aligned}$$

Since $W\subset \overline{B}$, then $W\cap g_{L-m+1}\cap \overline{B}=W\cap g_{L-m+1}$. Therefore, $P\left( C|W\cap g_{L-m+1}\cap \overline{B}\right) =P\left( C|W\cap g_{L-m+1}\right) =q_{L-m+1}$. According to Eq. (12), $P\left( W|g_{L-m+1}\cap \overline{B}\right) =\left( 1-p_b\right) ^{L-m+1}$. Since the randomness of the attack, the non-black network nodes are distributed between the network layers with the same proportions as between the layers’ sizes, causing $P\left( g_{L-m+1}|\overline{B}\right) =P\left( g_{L-m+1}\right)$. The probability $P\left( g_{L-m+1}\right)$ is the ratio between the size of layer $L-m+1$ which is $Z\left( Z-1\right) ^{L-m}$ and the network size which is approximately $\frac{Z}{Z-2}\left( Z-1\right) ^L$. Substituting these considerations into Eq. (41), and taking into account that between the stages *k* and $k+1$ of an attack, $p_b=\frac{k}{N}$, gives

42 $$\begin{aligned} Q_{L-m+1}&=\left( 1-\frac{k}{N}\right) ^{\frac{1}{Z-2}\left( \left( Z-1\right) ^m-Z+1\right) +L-m+1}\nonumber \\& \quad \cdot \left( Z-2\right) \left( \frac{1}{Z-1}\right) ^m. \end{aligned}$$

where $$m=1,2,\ \ldots \,L$$.

By definition, $Q_{L-m+1}$ is the probability of randomly attacking a white node in the $k+1$ stage of an attack that causes a complete avalanche of size $\frac{\left( Z-1\right) ^m-1}{Z-2}$.

IV. Probability of a complete avalanche of size $\frac{\left( Z-1\right) ^m-1}{Z-2}$ during an entire attack.

We define an indicator random variable $X_k$ for the event of a complete avalanche of size $\frac{\left( Z-1\right) ^m-1}{Z-2}$ in stage $k+1$ of an attack, and a random variable *X* that is the number of complete avalanches of size $\frac{\left( Z-1\right) ^m-1}{Z-2}$ during an entire attack until the network collapses, i.e. $X=\sum _{k=0}^{\frac{Z-2}{Z-1}N-1}X_k$. Therefore, the expected number of complete avalanches of size $\frac{\left( Z-1\right) ^m-1}{Z-2}$ during an attack until the network collapses is

43 $$\begin{aligned} E\left[ X\right]&=\sum _{k=0}^{\frac{Z-2}{Z-1}N-1} E\left[ X_k\right] \nonumber \\&= \sum _{k=0}^{\frac{Z-2}{Z-1}N-1}\left( 1-\frac{k}{N}\right) ^{\frac{1}{Z-2}\left( \left( Z-1\right) ^m-Z+1\right) +L-m+1}\nonumber \\&\quad \cdot \left( Z-2\right) \left( \frac{1}{Z-1}\right) ^m. \end{aligned}$$

To calculate this term, we approximate it by the following integral

44 $$\begin{aligned} E\left[ X\right]&=\left( Z-2\right) \left( \frac{1}{Z-1}\right) ^m\nonumber \\&\int _0^{\frac{Z-2}{Z-1}N-1}\left( 1-\frac{k}{N}\right) ^{\frac{1}{Z-2}\left( \left( Z-1\right) ^m-Z+1\right) +L-m+1}dk. \end{aligned}$$

By changing variables with the formula $u=1-\frac{k}{N}$ and performing some algebraic operations, we get

45 $$\begin{aligned} E\left[ X\right]&=\left( Z-2\right) \left( \frac{1}{Z-1}\right) ^m\nonumber \\&\int _1^{\frac{1}{Z-1}+\frac{1}{N}}u^{\frac{1}{Z-2}\left( \left( Z-1\right) ^m-Z+1\right) +L-m+1}\cdot \left( -N\right) du. \end{aligned}$$

Solving the integral and neglecting terms which are relatively very small, yields

46 $$\begin{aligned} E[X]&=\left( Z-2\right) \left( \frac{1}{Z-1}\right) ^mN\cdot \nonumber \\&\frac{1}{\frac{1}{Z-2}\left( \left( Z-1\right) ^m-Z+1\right) +L+2-m}. \end{aligned}$$

We rewrite Eq. (46) using an exponential term, and get

47 $$\begin{aligned} E\left[ X\right] =N&\left( Z-2\right) \left( \frac{1}{Z-1}\right) ^m\frac{1}{L}\cdot \nonumber \\&e^{-ln\left( 1+\frac{\frac{\left( Z-1\right) ^m}{Z-2}-\frac{Z-1}{Z-2}+2-m}{L}\right) }. \end{aligned}$$

Assume a random choice of one attack stage from the set of $\frac{Z-2}{Z-1}N$ attack stages. We denote by $S_m$ the event of a complete avalanche of size $\frac{\left( Z-1\right) ^m-1}{Z-2}$ occurs due to the node removal in the chosen stage. The probability of $S_m$ is the ratio of the expected number of complete avalanches of size $\frac{\left( Z-1\right) ^m-1}{Z-2}$ during an entire attack, calculated as $E\left[ X\right]$ in Eq. (47) above, and the number of the attack stages $\frac{Z-2}{Z-1}N$. Therefore, we have

48 $$\begin{aligned} P\left( S_m\right) & = \left( \frac{1}{Z-1}\right) ^{m-1}\frac{1}{L}\cdot \nonumber \\&e^{-ln\left( 1+\frac{\frac{\left( Z-1\right) ^m}{Z-2}-\frac{Z-1}{Z-2}+2-m}{L}\right) }. \end{aligned}$$

The exponent in Eq. (48) is dominated by the term $\left( Z-1\right) ^m$ compared to the other terms. Thus, the term $e^{-\ln \left( 1+\frac{\frac{\left( Z-1\right) ^m}{Z-2}-\frac{Z-1}{Z-2}+2-m}{L}\right) }$ approaches 1 when *m* is small relative to $\log _{Z-1}L$ and approaches 0 when *m* is large relative to $\log _{Z-1}L$, with a narrow transition region around $\log _{Z-1}L$. Therefore, we can approximate this term as 1 for values of *m* less than or equal to $\log _{Z-1}L$, and as 0 for values of *m* greater than $\log _{Z-1}L$. Applying this approximation in Eq. (48) yields the following distribution of a complete avalanche of size $\frac{\left( Z-1\right) ^m-1}{Z-2}$ $(m=1,2,3,\ldots ,L)$:

49 $$\begin{aligned} P\left( S_m\right) = {\left\{ \begin{array}{ll} \frac{1}{L}\cdot \frac{1}{\left( Z-1\right) ^{m-1}} &{} m\le \log _{Z-1}L\\ 0 &{} m>\log _{Z-1}L\\ \end{array}\right. } \end{aligned}$$

22 Probability of a complete avalanche of any size during an entire attack.

Assume a random choice of one attack stage from the set of $\frac{Z-2}{Z-1}N$ attack stages. We denote by $S_C$ the event of a complete avalanche that occurs due to the node removal in the chosen stage. The probability of $S_C$ is the sum of the probabilities of the events $S_m$. Using Eq. (49) accordingly gives:

$$\begin{aligned} P\left( S_C\right) =\sum _{m=1}^{\log _{Z-1}L}\frac{1}{L}\left( \frac{1}{Z-1}\right) ^{m-1}. \end{aligned}$$

Calculation of the previous series gives

50 $$\begin{aligned} P\left( S_C\right) =\frac{\frac{Z-1}{Z-2}}{L}\left( 1-\frac{1}{L}\right) . \end{aligned}$$

Under the assumption of a very large *L*, we neglect the term $\frac{1}{L}$ in the brackets, and obtain the final expression:

51 $$\begin{aligned} P\left( S_C\right) =\frac{\frac{Z-1}{Z-2}}{L}. \end{aligned}$$

#### Summary of theory

The considerations and calculations detailed above provide the following conclusions about the distribution of avalanche sizes in a random attack on a CT with *Z* neighbors: Assuming a very large *L*, the probability of a null avalanche occurring in a randomly chosen stage of an attack is given by (Eq. (32) above):

52 $$\begin{aligned} P\left( S_0\right) =1-\frac{\frac{Z-1}{Z-2}}{L}. \end{aligned}$$

The probability of a complete avalanche of size $\frac{\left( Z-1\right) ^m-1}{Z-2}$ $\left( m=1,2,3,\ldots ,L\right)$ occurring in a randomly chosen stage of an attack is given by (Eq. (49) above):

53 $$\begin{aligned} P\left( S_m\right) = {\left\{ \begin{array}{ll} \frac{1}{L}\cdot \frac{1}{\left( Z-1\right) ^{m-1}} &{} m\le \log _{Z-1}L\\ 0 &{} m>\log _{Z-1}L\\ \end{array}\right. } \end{aligned}$$

Assuming a very large *L*, the probability of a complete avalanche of any size occurring in a randomly chosen stage of an attack is given by (Eq. (51) above):

54 $$\begin{aligned} P\left( S_C\right) =\frac{\frac{Z-1}{Z-2}}{L}. \end{aligned}$$

Equations (52) and (54) present that the events $S_C$ of a complete avalanche in a randomly chosen stage of an attack and $S_0$ of a null avalanche in a randomly chosen stage of an attack are complementary events, where $P\left( S_C\right) +P\left( S_0\right) =1$. This leads to an interesting result, that during an attack on a CT with a very large *L*, either complete avalanches or null avalanches occur, and no incomplete avalanche occurs in either case.

**Figure 2:**
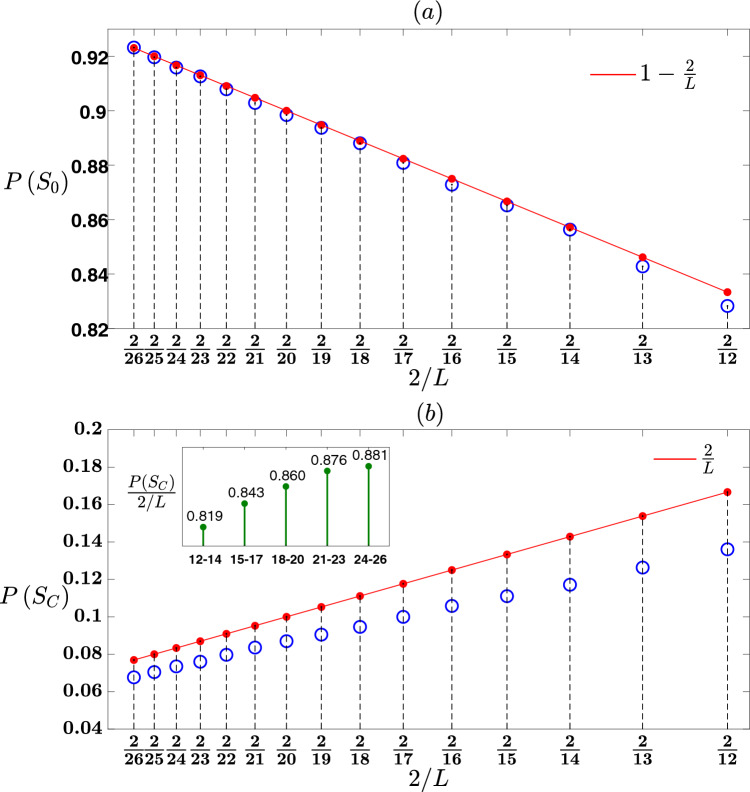
(**a**,**b**) Presentation of the avalanche-size distribution for a CT with $Z=3$ neighbors. (**a**) Presentation of $P\left( S_0\right)$ as predicted by theory and obtained by simulations, versus $\frac{2}{L}$. The x-axis scale marks correspond to $\frac{2}{26}, \frac{2}{25}, \frac{2}{24}\ldots , \frac{2}{12}$ representing CTs with $L=26,25,24\ldots , 12$ layers, respectively. At the top of each vertical dashed line, which corresponds to a specific scale mark of $\frac{2}{L}$, a red dot represents the value of $1-\frac{2}{L}$ as predicted by theory, and a blue circle represents the value of $P\left( S_0\right)$ as was obtained from simulations. The red line represents the graph of the line $1-\frac{2}{L}$. (**b**) Presentation of $P\left( S_C\right)$ as predicted by theory and obtained by simulations, versus $\frac{2}{L}$. At the top of each vertical dashed line, which corresponds to a specific scale mark of $\frac{2}{L}$, a red dot represents the value of $\frac{2}{L}$ as predicted by the theory, and a blue circle represents the value of $P\left( S_C\right)$ as was obtained from simulations. The red line represents the graph of the line $\frac{2}{L}$. Inset in panel (**b**) Bar graph showing the mean value of the ratio between $P\left( S_C\right)$ and $\frac{2}{L}$ for five subsets of *L* values: 12–14, 15–17, 18–20, 21–23 and 24–26. The averages were calculated over 1000 realizations.

### Simulations and results

Figure 2 demonstrates the validity of the theory for a CT with $Z=3$. For this CT the theory predicts as follows (following Eqs. (52)–(54)): Under the assumption of a very large *L*, the probability of a null avalanche event in a randomly chosen stage of an attack is

$$\begin{aligned} P\left( S_0\right) =1-\frac{2}{L}. \end{aligned}$$

The probability of a complete avalanche event of size $2^m-1$ $\left( m=1,2,3,\ldots ,L\right)$ in a randomly chosen stage of an attack is

$$\begin{aligned} P\left( S_m\right) = {\left\{ \begin{array}{ll} \frac{1}{L}\cdot \frac{1}{2^{m-1}} &{} m\le \log _2L\\ 0 &{} m>\log _2L\\ \end{array}\right. } \end{aligned}$$

Under the assumption of a very large *L*, the probability of a complete avalanche event in a randomly chosen stage of an attack is

$$\begin{aligned} P\left( S_C\right) =\frac{2}{L}. \end{aligned}$$

Also, for a CT with $Z=3$, the number of stages of an attack until the network is dismantled is $\frac{Z-2}{Z-1}N=\frac{N}{2}$.

The figure presents simulations results for a CT with $Z=3$ and $L=12, 13, 14,\ldots , 26$. In Fig. 2a the graph presents the probability of a null avalanche $P\left( S_0\right)$, as predicted by the theory and obtained by simulations, versus $\frac{2}{L}$. The x-axis scale marks of $\frac{2}{26}, \frac{2}{25}, \frac{2}{24},\ldots , \frac{2}{12}$ correspond to a CT with $L=26,25,24,\ldots , 12$, respectively. For each value of $\frac{2}{L}$, at the top of the vertical dashed line belongs to it a red dot represents the value of $1-\frac{2}{L}$ which is the probability $P\left( S_0\right)$ as predicted by the theory, and a blue circle represents the value of $P\left( S_0\right)$ was obtained from simulations and calculated as the average number of null avalanches in 1000 realizations that were simulated divided by the number of attack stages $\frac{N}{2}$. It is shown that as *L* increases from 12 to 26, i.e $\frac{2}{L}$ decreases from $\frac{2}{12}$ to $\frac{2}{26}$, the blue circles tend to coincide with the red dots. For $L=25$ and 26, there is indeed a good match between the blue circle and the red dot. This indicates that as *L* increases, $P\left( S_0\right)$ calculated from simulations approaches to the predicted value of $1-\frac{2}{L}$.

In Fig. 2b the graph presents the probability of a complete avalanche $P\left( S_C\right)$ as predicted by theory and obtained by simulations, versus $\frac{2}{L}$. As shown in panel $\left( a\right)$, for each $\frac{2}{L}$ value at the top of the vertical dashed line belongs to it, a red dot represents the value of $P\left( S_C\right)$ according to theory which is $\frac{2}{L}$, and a blue circle represents the value of $P\left( S_C\right)$ obtained from simulations, which was calculated again as the average of the number of complete avalanches in 1000 realizations divided by the number of attack stages $\frac{N}{2}$. It is observed that as *L* increases from 12 to 26, the blue circles approach the red dots. The inset of Fig. 2b provides a more detailed illustration of this trend. For each of the fifteen CTs simulated, with *L* values ranging from 12 to 26, the ratio of $P\left( S_C\right)$ as obtained from the simulations and $\frac{2}{L}$ was calculated. The *L* values were then divided into five subsets: 12–14, 15–17, 18–20, 21–23, and 24–26. The mean of the ratio between $P\left( S_C\right)$ and $\frac{2}{L}$ was calculated for each subset, considering the three members of the subset. For example, for the subset of the *L* values 12, 13 and 14, the following mean was calculated: $\frac{1}{3}\cdot \left( \frac{P\left( S_C\right) _{12}}{2/12}+\frac{P\left( S_C\right) _{13}}{2/13}+\frac{P\left( S_C\right) _{14}}{2/14}\right)$. Here, $P\left( S_C\right) _{12}$ represents the probability of a complete avalanche as was obtained from simulations on the CT with $L=12$, and so on. The inset of Fig. 2b shows a bar graph, where each of the five subsets is represented by one bar. The height of each bar corresponds to the mean of the ratio between $P\left( S_C\right)$ and $\frac{2}{L}$ calculated for the subset it represents, and the value of this ratio is marked on top of the bar. Recall that according to our theory, as *L* increases, $P\left( S_C\right)$ becomes greater and tends to $\frac{2}{L}$, that is $\frac{P\left( S_C\right) }{2/L}$ becomes greater and tends to 1. The bar graph shows that as *L* increases within the subsets, the mean of the ratio $\frac{P\left( S_C\right) }{2/L}$ becomes greater, i.e. from 0.819 for the the subset $12-14$ to 0.881 for the subset $24-26$, approaching towards the value of 1 as predicted by the theory. It is worth noting that to obtain values close to this limit, a network with an extremely large size (tending towards infinity mathematically) must be simulated. Another confirmation of the validity of the theory pertains to the prediction of the probability of a complete avalanche of size $2^m-1$ as $\frac{1}{L}\cdot \frac{1}{2^{m-1}}$ if $m\le \log _2L$ and 0 if $m>\log _2L$. For instance, consider the first subset where *L* varies between 12 to 14, and the respective $\log _2L$ values range from $\log _212=3.58$ to $\log _214=3.81$. The theory predicts that the probability of a complete avalanche of size $2^m-1$ is $\frac{1}{L}\cdot \frac{1}{2^{m-1}}$ if $m\le 3$ and 0 if $m>3$. Therefore, for this subset, the probability of a complete avalanche of any size $P\left( S_C\right)$ is predicted to be $\frac{1}{L}\left( \frac{1}{2^{1-1}}+\frac{1}{2^{2-1}}+\frac{1}{2^{3-1}}\right) =\frac{1.75}{L}$, and $\frac{P\left( S_C\right) }{2/L}$ is predicted to be 0.875. The inset of Fig. 2b illustrates that the simulation’s result of $\frac{P\left( S_C\right) }{2/L}$ for this subset is 0.819, which corresponds to $93.6\%$ of the predicted value 0.875. Similarly, let us consider the last subset where *L* varies between 24 and 26, and $\log _2L$ varies between $\log _224=4.58$ and $\log _225=4.64$. According to the theory, the probability of a complete avalanche of size $2^m-1$ for this subset is $\frac{1}{L}\cdot \frac{1}{2^{m-1}}$ if $m\le 4$ and 0 if $m>4$. Therefore, the probability of a complete avalanche of any size, $P\left( S_C\right)$, is predicted to be $\frac{1}{L}\left( \frac{1}{2^{1-1}}+\frac{1}{2^{2-1}}+\frac{1}{2^{3-1}}+\frac{1}{2^{4-1}}\right) =\frac{1.875}{L}$, and consequently, $\frac{P\left( S_C\right) }{2/L}$ is predicted to be 0.9375. The inset of Fig. 2b shows that the simulation’s result of $\frac{P\left( S_C\right) }{2/L}$ for this subset is indeed 0.881, which is $94\%$ of the predicted value 0.9375. The results for the other subsets can be similarly analyzed.

**Figure 3:**
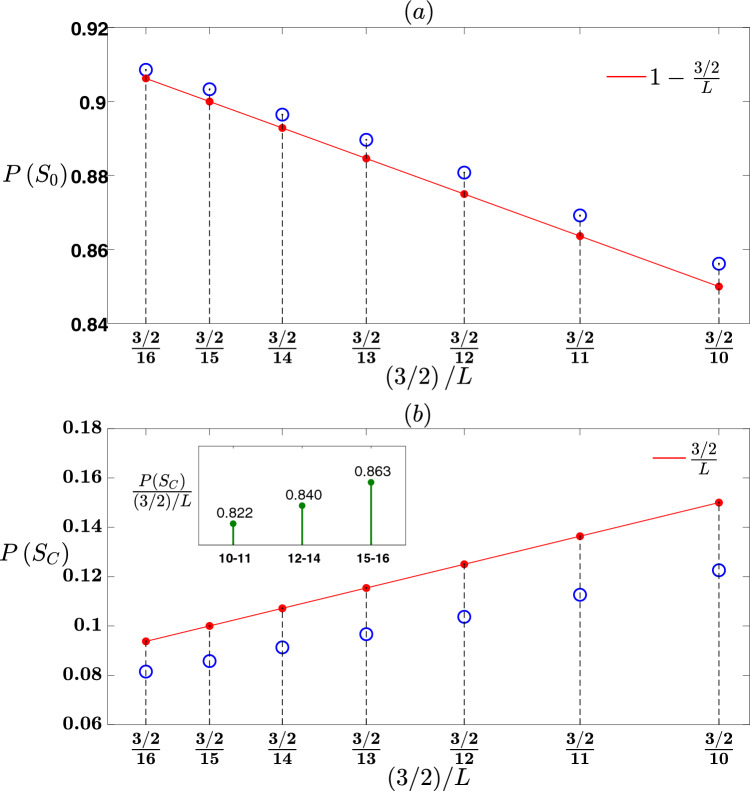
(**a**,**b**) Presentation of the avalanche-size distribution for a CT with $Z=4$ neighbors. (**a**) Presentation of $P\left( S_0\right)$ as predicted by theory and observed in simulations, versus $\frac{3/2}{L}$. The x-axis scale marks correspond to $\frac{3/2}{16}, \frac{3/2}{15}, \frac{3/2}{14}\ldots , \frac{3/2}{10}$ representing CTs with $L=16,15,14\ldots , 10$ layers, respectively. At the top of each vertical dashed line, which corresponds to a specific scale mark of $\frac{3/2}{L}$, a red dot represents the value of $1-\frac{3/2}{L}$ as predicted by theory, and a blue circle represents the value of $P\left( S_0\right)$ obtained from simulations. The red line represents the graph of the line $1-\frac{3/2}{L}$. (**b**) Presentation of $P\left( S_C\right)$ as predicted by theory and observed in simulations, versus $\frac{3/2}{L}$. At the top of each vertical dashed line, which corresponds to a specific scale mark of $\frac{3/2}{L}$, a red dot represents the value of $\frac{3/2}{L}$ as predicted by theory, and a blue circle represents the value of $P\left( S_C\right)$ obtained from simulations. The red line represents the graph of the line $\frac{3/2}{L}$. Inset in panel (**b**) Bar graph showing the mean value of the ratio between $P\left( S_C\right)$ and $\frac{3/2}{L}$ for three subsets of *L* values: 10–11, 12–14 and 15–16. The averages were calculated over 1000 realizations.

Figure 3 provides further evidence of the validity of the theory for a CT with $Z=4$. The theory predicts the following: Assuming a very large *L*, the probability of the event of a null avalanche in a randomly chosen stage of an attack is given by

$$\begin{aligned} P\left( S_0\right) =1-\frac{3/2}{L}. \end{aligned}$$

The probability of the event of a complete avalanche of size $\frac{3^m-1}{2}$ $\left( m=1,2,3,\ldots ,L\right)$ in a randomly chosen stage of an attack is given by

$$\begin{aligned} P\left( S_m\right) = {\left\{ \begin{array}{ll} \frac{1}{L}\cdot \frac{1}{3^{m-1}} &{} m\le \log _3L\\ 0 &{} m>\log _3L\\ \end{array}\right. } \end{aligned}$$

Assuming a very large *L*, the probability of the event of a complete avalanche in a randomly chosen stage of an attack is

$$\begin{aligned} P\left( S_C\right) =\frac{3/2}{L}. \end{aligned}$$

For a CT with $Z=4$, the number of stages of an attack until the network is dismantled is $\frac{Z-2}{Z-1}N=\frac{2}{3}N$.

The figure presents simulations results for a CT with $Z=4$ and $L=10, 11, 12,\ldots , 16$. Its pattern is similar to Fig. 2. In Fig. 3a the graph presents the probability of a null avalanche $P\left( S_0\right)$ as predicted by the theory and obtained by simulations, versus $\frac{3/2}{L}$. The scale marks of x-axis that are $\frac{3/2}{16}, \frac{3/2}{15}, \frac{3/2}{14}\ldots , \frac{3/2}{10}$ correspond to a CT with $L=16,15,14\ldots , 10$, respectively. For each value of $\frac{3/2}{L}$, at the top of the vertical dashed line belongs to it a red dot represents the value of $1-\frac{3/2}{L}$ which is the probability of a null avalanche $P\left( S_0\right)$ as predicted by the theory, and a blue circle represents the value of $P\left( S_0\right)$ as was obtained from simulations and calculated as the average number of null avalanches in 1000 realizations that were simulated divided by the number of attack stages $\frac{2}{3}N$. It is shown that as *L* increases from 10 to 16, i.e $\frac{3/2}{L}$ decreases from $\frac{3/2}{10}$ to $\frac{3/2}{16}$, the blue circles tend to coincide with the red dots. For $L=15$ and 16, there is indeed a good match between the blue circles and the red dots. This indicates that as *L* increases, $P\left( S_0\right)$ calculated from simulations approaches to the predicted value of $1-\frac{3/2}{L}$. In Fig. 3b the graph presents the probability of a complete avalanche $P\left( S_C\right)$, as predicted by theory and obtained by simulations versus $\frac{3/2}{L}$. For each $\frac{3/2}{L}$ value, at the top of the vertical dashed line belongs to it a red dot represents the value of $P\left( S_C\right)$ according to theory which is $\frac{3/2}{L}$, and a blue circle represents the value of $P\left( S_C\right)$ obtained from simulations, which was calculated again as the average of the number of complete avalanches in 1000 realizations divided by the number of attack stages $\frac{2}{3}N$. It is observed that as *L* increases from 10 to 16, the blue circles approaches the red dots. The inset of Fig. 3b provides a more detailed illustration of this trend. For each of the seven CTs simulated, the ratio of $P\left( S_C\right)$ obtained from the simulations and $\frac{3/2}{L}$ was calculated. The *L* values were then divided to three subsets: 10–11, 12–14 and 15–16. The mean of the ratio between $P\left( S_C\right)$ and $\frac{3/2}{L}$ was calculated for each subset, considering the members of the subset. The inset of Fig. 3b shows a bar graph, where each of the three subsets is represented by one bar. The height of each bar corresponds to the mean of the ratio between $P\left( S_C\right)$ and $\frac{3/2}{L}$ calculated for the subset it represents, and the value of this ratio is marked on top of the bar. Recall that according to our theory, as *L* increases, $P\left( S_C\right)$ becomes greater and tends to $\frac{3/2}{L}$, that is $\frac{P\left( S_C\right) }{\frac{3/2}{L}}$ becomes greater and tends to 1. The bar graph shows that as *L* increases within the subsets, the mean ratio of $\frac{P\left( S_C\right) }{\frac{3/2}{L}}$ becomes greater, i.e. from 0.822 for the subset 10–11 to 0.863 for the subset 14–16, approaching towards the value of 1. Another confirmation of the validity of the theory pertains to the prediction of the probability of a complete avalanche of size $\frac{3^m-1}{2}$ as $\frac{1}{L}\frac{1}{3^{m-1}}$ if $m\le \log _3L$ and 0 if $m>\log _3L$. We consider, for example, the last subset where *L* varies between 15 to 16, and the respective $\log _3L$ values range from $\log _315=2.46$ to $\log _316=2.52$. The theory predicts that the probability of a complete avalanche of size $\frac{3^m-1}{2}$, is $\frac{1}{L}\cdot \frac{1}{3^{m-1}}$ if $m\le 2$ and 0 if $m>2$. Therefore, for this subset, the probability of a complete avalanche of any size $P\left( S_C\right)$ is predicted to be $\frac{1}{L}\left( \frac{1}{3^{1-1}}+\frac{1}{3^{2-1}}\right) =\frac{4/3}{L}$, and $\frac{P\left( S_C\right) }{\left( 3/2\right) /L}$ is predicted to be 0.889. The inset of Fig. 3b illustrates that the simulation’s result of $\frac{P\left( S_C\right) }{\left( 3/2\right) /L}$ for this subset is 0.863, which corresponds to $97\%$ of the predicted value 0.889. Similarly are the results for the other subsets too.

## Discussion and summary

In this work, we applied a method of analyzing attacks on networks from the microscopic perspective, considering the impact and contribution of each node removal during an attack, to the fragmentation (immunization) of the network. This approach is in contrast to the traditional methods that analyze attacks on networks from a macroscopic perspective, characterizing the attack and its effectiveness using parameters related to the entire attack, such as the critical probability of percolation occurrence $p_c$ and the size of the giant component. We found that in the case of a random attack on a CT with *Z* neighbors and a very large number of layers *L*, if we randomly choose one attack stage from the set of $\frac{Z-2}{Z-1}N$ attack stages (which is the number of node removals required to dismantle the network), the probability that the node removal in this chosen stage causes a null avalanche is $1-\frac{\frac{Z-1}{Z-2}}{L}$. Considering the assumption of a very large *L*, this probability is not significantly smaller than 1. Consequently, we conclude that almost all the nodes that are removed during the attack cause null avalanche, indicating that these nodes were not connected to the giant component even before they were attacked. This leads to the interesting finding, that in our case the majority of node removals during an attack have no effect or contribution to the destruction of the network. On the other hand, we found that the probability that a node removal in a randomly chosen stage of an attack causes a complete avalanche is $\frac{\frac{Z-1}{Z-2}}{L}$. That is, the event of a complete avalanche is the complement of the event of a null avalanche. This leads to the other interesting conclusion, that besides the very large portion of null avalanches that occur during an attack, there is also a small fraction of non-null avalanches, but of the type of complete avalanche only. The other type of incomplete avalanche does not occur at all during the attack. The complete avalanches that do occur during an attack are the cause of the network collapse.

We hope that these findings will deepen our insights of how networks collapse and serve as a tool for improving network immunization processes.

We also note that the current work could serve as the foundation for future studies on the avalanche-size distribution of networks under attack. These studies could investigate networks with topologies other than the CT topology, such as scale-free networks, small-world networks and random networks. Additionally, they could explore different types of attacks, including targeted attacks, local attacks, etc., and compare the results with those obtained in CT networks. In general, the current work could serve as a foundation for researching attacks on networks from a micro perspective. This involves the impact of the local stages of an attack on some local parts of the targeted network and characterizing how networks dismantle step by step during an attack. An interesting issue of investigation would be a comparison between the micro and macro perspectives of attacks on networks. This field of research has substantial potential and significant implications.

## Supplementary Information

Supplementary Information.

## Data Availability

The datasets used and/or analysed during the current study are available from the corresponding author on reasonable request.
