# Supplementary material for: Avalanche-size distribution of Cayley tree
Source: Sci Rep. 2023 Jul 13;13:11311. doi: 10.1038/s41598-023-38332-1 (PMC10345117; doi:10.1038/s41598-023-38332-1)
Supplement: Supplementary file 1 — Supplementary Information. [file 41598_2023_38332_MOESM1_ESM.pdf]

Supplementary Information for "Avalanche-size distribution of Cayley tree"

(Dated: May 23, 2023)

I. The identity $\frac{1}{L-j+1}=\frac{1}{L+1}\sum_{m=0}^{\infty}\frac{j^m}{(L+1)^m}$

We begin with the following

$$\frac{L+1}{L-j+1}=\frac{1}{1-\frac{j}{L+1}}. \tag{A1}$$

We assume a very large L, and applying the series $\frac{1}{1-x}=1+x+x^2+x^3+\ldots$, so we get

$$\frac{1}{1-\frac{j}{L+1}}=1+\frac{j}{L+1}+\left(\frac{j}{L+1}\right)^2+\left(\frac{j}{L+1}\right)^3+\ldots$$
$$=\sum_{m=0}^{\infty}\left(\frac{j}{L+1}\right)^m. \tag{A2}$$

In summary we get

$$\frac{L+1}{L-j+1}=\sum_{m=0}^{\infty}\left(\frac{j}{L+1}\right)^m, \tag{A3}$$

that is

$$\frac{1}{L-j+1}=\frac{1}{L+1}\sum_{m=0}^{\infty}\frac{j^m}{(L+1)^m}, \tag{A4}$$

as required.

II. The identity $\sum_{j=1}^{L+1}\frac{(Z-1)^j-1}{j}=(Z-1)^{L+1}\sum_{m=0}^{\infty}\frac{1}{(L+1)^{m+1}}\sum_{j=0}^{\infty}\frac{j^m}{(Z-1)^j}$

We change variables from j to x according to the formula $j=L-x+1$. Therefore, $x=L-j+1$, and $j=1$ is equivalent to $x=L$ and $j=L+1$ is equivalent to $x=0$. Therefore we get

$$\sum_{j=1}^{L+1}\frac{(Z-1)^j-1}{m}=\sum_{x=L}^{0}\frac{(Z-1)^{L+1-x}-1}{L+1-x}. \tag{B1}$$

We rewrite the right-hand-side of the previous expression with the variable j, and perform the identity $\frac{1}{L-j+1}=\frac{1}{L+1}\sum_{m=0}^{\infty}\frac{j^m}{(L+1)^m}$. Thus we get

$$\sum_{j=L}^{0}\frac{(Z-1)^{L-j+1}-1}{L-j+1}$$
$$=\sum_{j=0}^{L}\frac{1}{L+1}\sum_{m=0}^{\infty}\frac{j^m}{(L+1)^m}\left((Z-1)^{L-j+1}-1\right)$$
$$\approx\sum_{j=0}^{L}\frac{1}{L+1}\sum_{m=0}^{\infty}\frac{j^m}{(L+1)^m}\cdot(Z-1)^{L-j+1}, \tag{B2}$$

where the last approximation is due to the assumption of $L>>1$. The last term can be rewritten as follows –

$$(Z-1)^{L+1}\sum_{m=0}^{\infty}\frac{1}{(L+1)^{m+1}}\sum_{j=0}^{L}\frac{j^m}{(Z-1)^j}$$
$$=(Z-1)^{L+1}\sum_{m=0}^{\infty}\frac{1}{(L+1)^{m+1}}\cdot$$
$$\left(\sum_{j=0}^{\infty}\frac{j^m}{(Z-1)^j}-\sum_{j=L+1}^{\infty}\frac{j^m}{(Z-1)^j}\right). \tag{B3}$$

Since $L>>1$, we approximate the term $\sum_{j=L+1}^{\infty}\frac{j^m}{(Z-1)^j}$ to 0. Therefore we get

$$(Z-1)^{L+1}\sum_{m=0}^{\infty}\frac{1}{(L+1)^{m+1}}\sum_{j=0}^{\infty}\frac{j^m}{(Z-1)^j}, \tag{B4}$$

as required.

III. The series $\sum_{j=0}^{\infty}\frac{j^0}{(Z-1)^j}=\frac{Z-1}{Z-2}$, $\sum_{j=0}^{\infty}\frac{j^1}{(Z-1)^j}=\frac{Z-1}{(Z-2)^2}$ and $\sum_{j=0}^{\infty}\frac{j^2}{(Z-1)^j}=\frac{Z(Z-1)}{(Z-2)^3}$

We begin with the familiar geometric series with the ratio x where $x<1$, which is

$$\sum_{j=0}^{\infty}x^j=\frac{1}{1-x}. \tag{C1}$$

Substituting into the previous equation $x=\frac{1}{Z-1}$ gives the first required series

$$\sum_{j=0}^{\infty}\left(\frac{1}{Z-1}\right)^j=\frac{1}{1-\frac{1}{Z-1}}=\frac{Z-1}{Z-2} \tag{C2}$$

Differentiating both sides of Eq. $(C1)$ term by term gives

$$\sum_{j=0}^{\infty}jx^{j-1}=\frac{1}{(1-x)^2}. \tag{C3}$$

substituting $x=\frac{1}{Z-1}$ into Eq. $(C3)$ gives

$$\sum_{j=0}^{\infty}j\left(\frac{1}{Z-1}\right)^{j-1}=\frac{1}{\left(1-\frac{1}{Z-1}\right)^2}. \tag{C4}$$

Performing some algebraic operations gives the second required series

$$\sum_{j=0}^{\infty}\frac{j}{(Z-1)^j}=\frac{Z-1}{(Z-2)^2} \tag{C5}$$

The first term of the series in the LHS of Eq. $(C3)$ equals 0, so it can be rewritten as follows

$$\sum_{j=1}^{\infty} j x^{j-1} = \frac{1}{(1-x)^2}. \quad \text{(C6)}$$

Changing the variable j in Eq. $(C6)$ to k with the rule of $j = k+1$ gives

$$\sum_{k=0}^{\infty} (k+1)\, x^k = \frac{1}{(1-x)^2}. \quad \text{(C7)}$$

Rearranging this equation Considering that $\sum_{k=0}^{\infty} x^k = \frac{1}{1-x}$, yields

$$\sum_{k=0}^{\infty} k^2 x^{k-1} = \frac{1+x}{(1-x)^3} \quad \text{(C8)}$$

Substituting $x = \frac{1}{Z-1}$ into Eq. $(C8)$, performing some algebraic operations and replacing formally again the variable k to j, gives the third required identity

$$\sum_{j=0}^{\infty} \frac{j^2}{(Z-1)^j} = \frac{Z(Z-1)}{(Z-2)^3} \quad \text{(C9)}$$

IV. Changing base of expansion

We have presented the equality between

$$N\left(\frac{1}{L+1} + \frac{\frac{1}{Z-2}}{(L+1)^2} + \frac{\frac{Z}{(Z-2)^2}}{(L+1)^3} + O\left(\frac{1}{(L+1)^4}\right)\right), \quad \text{(D1)}$$

and

$$N\left(\frac{1}{L} + \frac{\frac{3-Z}{Z-2}}{L^2} + \frac{\frac{Z^2-5Z+8}{2(Z-2)^2}}{L^3} + O\left(\frac{1}{L^4}\right)\right). \quad \text{(D2)}$$

The change of the base of the expansion is implemented as folows – we begin with the followings

$$\frac{1}{n-1} = \frac{1}{n} \cdot \frac{1}{1-\frac{1}{n}}. \quad \text{(D3)}$$

We apply the series $\frac{1}{1-x} = 1 + x + x^2 + x^3 + \ldots$. Therefore we get

$$\frac{1}{n-1} = \frac{1}{n} \cdot \left(1 + \frac{1}{n} + \frac{1}{n^2} + \frac{1}{n^3}\right). \quad \text{(D4)}$$

We also write

$$\frac{1}{(n-1)^2} = \frac{1}{n^2} \cdot \frac{1}{\left(1-\frac{1}{n}\right)^2}. \quad \text{(D5)}$$

since the followings

$$\frac{1}{(1-x)^2} = \frac{d}{dx}\left(\frac{1}{1-x}\right) = 1 + 2x + 3x^2 + 4x^3, \quad \text{(D6)}$$

then

$$\frac{1}{(n-1)^2} = \frac{1}{n^2} \cdot \left(1 + \frac{2}{n} + \frac{3}{n^2} + \frac{4}{n^3}\right). \quad \text{(D7)}$$

We write

$$\frac{1}{(n-1)^3} = \frac{1}{n^3} \cdot \frac{1}{\left(1-\frac{1}{n}\right)^3}. \quad \text{(D8)}$$

since

$$\frac{1}{(1-x)^3} = \frac{1}{2}\frac{d}{dx}\left(\frac{1}{(1-x)^2}\right) = 1 + 3x + 6x^2 + 10x^3, \quad \text{(D9)}$$

therefore we get

$$\frac{1}{(n-1)^3} = \frac{1}{n^3} \cdot \left(1 + \frac{3}{n} + \frac{6}{n^2} + \frac{10}{n^3}\right). \quad \text{(D10)}$$

We present the equation

$$\begin{aligned}
&\frac{a_1}{n} + \frac{a_2}{n^2} + \frac{a_3}{n^3} + \cdots = \frac{b_1}{n-1} + \frac{b_2}{(n-1)^2} + \frac{b_3}{(n-1)^3} + \ldots \\
=&\frac{b_1}{n} \cdot \left(1 + \frac{1}{n} + \frac{1}{n^2} + \ldots\right) + \frac{b_2}{n^2} \cdot \left(1 + \frac{2}{n} + \frac{3}{n^2} + \ldots\right) \\
&+ \frac{b_3}{n^3} \cdot \left(2 + \frac{3}{n} + \frac{6}{n^2} + \ldots\right) + \cdots = \\
=&\frac{b_1}{n} + \frac{b_1 + b_2}{n^2} + \frac{b_1 + 2b_2 + 2b_3}{n^3} + \ldots . \quad \text{(D11)}
\end{aligned}$$

By a comparison of coefficients we get

$$\begin{aligned}
&b_1 = a_1 \\
&b_1 + b_2 = a_2 \quad \rightarrow \quad b_2 = a_2 - a_1 \\
&b_1 + 2b_2 + 2b_3 = a_3 \quad \rightarrow \quad b_3 = \frac{1}{2}(a_3 - 2a_2 + a_1). \quad \text{(D12)}
\end{aligned}$$

In our case, $a_1 = 1$, $a_2 = \frac{1}{Z-2}$ and $a_3 = \frac{Z}{(Z-2)^2}$. Calculating the b's coefficients according to the transformations in Eq. $(D12)$, gives that $b_1 = 1$, $b_2 = \frac{3-Z}{Z-2}$ and $b_3 = \frac{Z^2-5Z+8}{2(Z-2)^2}$ as required.
